# Comparative transcriptomics of Entelegyne spiders (Araneae, Entelegynae), with emphasis on molecular evolution of orphan genes

**DOI:** 10.1371/journal.pone.0174102

**Published:** 2017-04-05

**Authors:** David E. Carlson, Marshal Hedin

**Affiliations:** 1 Department of Biology, San Diego State University, San Diego, California, United States of America; 2 Department of Ecology & Evolution, Stony Brook University, Stony Brook, New York, United States of America; Scientific Research Centre of the Slovenian Academy of Sciences and Art, SLOVENIA

## Abstract

Next-generation sequencing technology is rapidly transforming the landscape of evolutionary biology, and has become a cost-effective and efficient means of collecting exome information for non-model organisms. Due to their taxonomic diversity, production of interesting venom and silk proteins, and the relative scarcity of existing genomic resources, spiders in particular are excellent targets for next-generation sequencing (NGS) methods. In this study, the transcriptomes of six entelegyne spider species from three genera (*Cicurina travisae*, *C*. *vibora*, *Habronattus signatus*, *H*. *ustulatus*, *Nesticus bishopi*, and *N*. *cooperi*) were sequenced and *de novo* assembled. Each assembly was assessed for quality and completeness and functionally annotated using gene ontology information. Approximately 100 transcripts with evidence of homology to venom proteins were discovered. After identifying more than 3,000 putatively orthologous genes across all six taxa, we used comparative analyses to identify 24 instances of positively selected genes. In addition, between ~ 550 and 1,100 unique orphan genes were found in each genus. These unique, uncharacterized genes exhibited elevated rates of amino acid substitution, potentially consistent with lineage-specific adaptive evolution. The data generated for this study represent a valuable resource for future phylogenetic and molecular evolutionary research, and our results provide new insight into the forces driving genome evolution in taxa that span the root of entelegyne spider phylogeny.

## Introduction

The rise of high throughput sequencing technologies (also known as next-generation sequencing, hereafter NGS) has created new research opportunities in many fields of biology, including evolutionary biology and systematics (e.g., [1,2]). For non-model organisms, shotgun sequencing of a transcriptome can be a useful and cost-effective means of gaining insight into genome-wide biological processes ([3]). One way that transcriptomic data have been used to study molecular and organismal evolution is through comparative analyses of sequences from multiple taxa. Comparative transcriptomics has been used, for example, to detect positive selection in genomes ([4,5]), estimate transcriptome-wide rates of molecular evolution ([6]), and resolve difficult phylogenetic questions (e.g., [2,7–9]).

Another area of research that has greatly benefited from the availability of NGS data is the study of orphan genes. Orphan genes are genes that are detected only in a particular lineage and lack recognizable protein-coding homologs in other taxa ([10]). The relative ease with which genomic data can be collected for non-model organisms is expanding our ability to identify and analyze the evolutionary dynamics of orphan genes ([11–13]). These taxonomically restricted genes are often thought to be implicated in lineage-specific adaptation to unique environmental conditions ([10]) and are thus of major interest to researchers. While examples of likely adaptive orphan genes have been identified ([14–16]), generalizations regarding the origin and maintenance of orphan genes have heretofore been limited by the availability of genomic or transcriptomic data.

**Fig 1:**
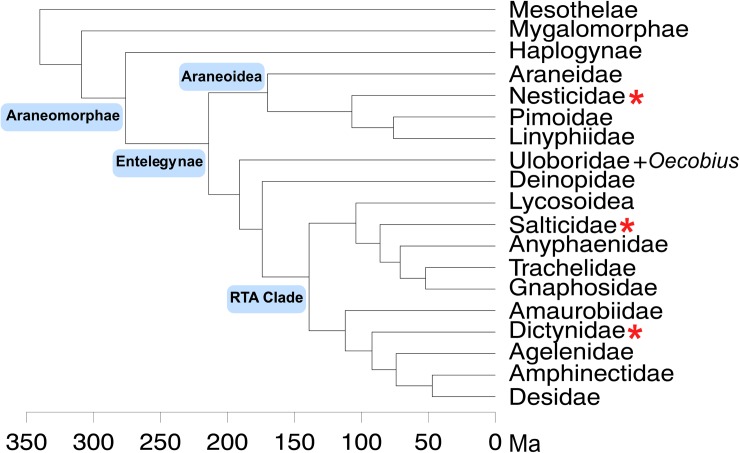
Time-calibrated backbone spider phylogeny. Modified from [9], this phylogeny shows the placement of major clades. Families containing taxa used in this study are highlighted with red asterisks.

Entelegynae is a clade of spiders that have traditionally been united by a number of shared derived morphological features, such as highly modified and complex male pedipalps ([17]). With more than 38,000 described species ([18]), the entelegynes include many of the most species-rich spider families, such as wolf spiders (Lycosidae), jumping spiders (Salticidae), sheat weavers (Linyphiidae), and orb weavers (Araneidae). Substantial research effort has focused on elucidating entelegyne phylogeny (e.g., [9,19–22]). Two of the largest entelegyne clades include the “RTA clade,” where males possess a palpal knob called the retrolateral tibial apophysis, and the Araneoidea, which consists of the ecribellate orb weavers and kin ([17,18]). Phylogenomic data estimate the age of Entelegynae as roughly 154–290 MA, the age of Araneoidea as 114–233 MA, and the age of the RTA clade as 83–201 MA (see Fig 1**; [9]**). Although spiders produce many unique silk and venom proteins, available genomic resources for spiders—and arachnids more broadly—remain limited and are not commensurate with the rich taxonomic diversity of this clade. While the first two spider genomes have been published ([23]) and spider-specific orthologous gene models are now available ([9]), large-scale, well-annotated genetic data remain unavailable for most spider taxa. As such, members of this understudied taxonomic group make excellent subjects for both molecular evolutionary research and increased DNA sequencing efforts.

In recent years, a number of studies utilizing NGS data have aimed to reveal information about the evolution of entelegyne spiders; however, much of this research focused on the evolution of a narrow set of proteins. For example, multiple transcriptomic studies characterizing spider venom [24–28]) and silk proteins ([29–32]) have been published. Conversely, studies that expand their scope to a broader array of genomic sequences have focused primarily on a fairly small phylogenetic range of taxa. Comparative transcriptomics have been used, for example, to investigate the evolution of sociality in three *Stegodyphus* species ([33]), and patterns of selection on ecomorphological diversification in Hawaiian *Tetragnatha* ([34,35]). Only very recently (e.g., [36]) have researchers begun using NGS data to examine patterns of genome-wide molecular evolution across divergent entelegyne taxa.

Six species representing three congeneric pairs of entelegyne genera were chosen for transcriptome assembly and analysis. These genera represent both the Araneoidea and RTA clades, and differ in ecology. *Nesticus* (Nesticidae) and *Cicurina* (Dictynidae) prefer sheltered microhabitats, such as in caves or under rocks in forests ([37,38]), while *Habronattus* jumping spiders (Salticidae) are active open-habitat visual hunters ([39,40]). *Cicurina* and *Habronattus* are both members of the RTA clade, while *Nesticus* is found within Araneoidea. The use of these relatively divergent taxa, which span the root of the entelegyne phylogeny, in conjunction with our congeneric pair sampling strategy enabled investigation of patterns of transcriptome composition and rates of molecular evolution both within and among entelegyne spider lineages. After *de novo* assembling each transcriptome, assessing the quality and completeness of each assembly, and performing functional annotation, we estimated substitution rates among putatively-orthologous genes in order to detect signatures of positive selection. In addition, we used the presence of novel coding sequences shared among congeneric pairs to identify lineage-specific orphan genes as well as undescribed genes likely present in a variety of entelegyne species. Our results provide evidence for a relatively low rate of positive selection among more than 3,300 single-copy orthologs. In addition, we find that many of the unique, uncharacterized orphan genes in each transcriptome exhibit elevated rates of substitution likely due to a combination of relaxed functional constraint in some cases and positive selection in others. These results are consistent with the hypothesis that some orphan genes are involved in lineage-specific adaptation.

## Materials and methods

### RNA extraction and sequencing

RNA was extracted from field-collected specimens preserved in RNAlater using RNeasy minikits (Qiagen, Valencia, CA), and shipped to the Genomic Services Lab at HudsonAlpha (www.hudsonalpha.org) where non-normalized cDNA libraries were sequenced on an Illumina Hiseq 2000 platform. As detailed in [41], cave-dwelling *Cicurina* were collected under a permit from the US Fish & Wildlife Service, issued to Cyndee Watson. *Habronattus* and *Nesticus* samples were collected from roadside right-of-way habitats in California and North Carolina where specific permissions were not required. *Habronattus* and *Nesticus* specimens were sequenced using 50 bp paired-end reads, while *Cicurina* specimens were sequenced using 100 bp paired-end reads. S1 Table summarizes the specimens/tissues used in each RNA extraction and the number of raw Illumina reads generated for each species.

### *De novo* transcriptome assembly

FastQC version 0.10.1 ([42]) was used to calculate read quantity, Phred scores, and to confirm quality of the raw reads prior to assembly. The perl script Trim Galore! (http://www.bioinformatics.babraham.ac.uk/projects/trim_galore/) was used to remove low quality sequences and trim Illumina adaptors. Sequences less than 30 base pairs in length, as well as those consisting of greater than one percent ambiguity characters, were removed with PRINSEQ Lite ([43]). Raw reads passing quality control were assembled *de novo* into transcripts using Trinity ([44]) with the default k-mer size of 25 and minimum transcript length set to 200 bp. Briefly, Trinity uses overlapping k-mers to assemble contigs, which are grouped together into clusters likely stemming from alternatively spliced isoforms of the same gene or products of closely related paralogs. Then, De Bruijn graphs are built for each cluster and used to reconstruct individual transcripts for each isoform and paralogous gene ([44]).

DeconSeq ([45]) was used to remove likely contaminant sequences. Reads were mapped back to assembled transcripts using Bowtie ([46]) in RSEM ([47]) to estimate abundance levels. To remove likely assembly errors, low abundance transcript outliers (i.e., individual isoform transcripts whose expression levels represented less than one percent of the overall expression for that gene) were filtered out of the assembly.

### Assembly summary statistics and completeness assessment

After quality filtering, Trinity and the python script sizecutter.py ([48]) were used to calculate a variety of assembly summary statistics. Read coverage depth was calculated with BEDtools using the genomeCoverageBed program ([49]). Read coverage is dependent on the level of expression for a given transcript, which varies greatly among sequences. Because of this, mean read coverage is expected to be inconsistent across different parts of the transcriptome ([44,50]). As such, we computed both the mean coverage and the proportion of each transcriptome with three different levels of read coverage.

The completeness and quality of each assembled transcriptome was evaluated using several procedures. The first measure was the Core Eukaryotic Genes Mapping Approach (CEGMA; [51,52]) which attempts to quantify the relative gene content of a particular set of sequences. This involves mapping assemblies to 248 core eukaryotic genes (CEGs) that have been found to be highly conserved and present in low copy number across six model eukaryotic organisms. To complement CEGMA analyses, we downloaded a recently published dataset consisting of hidden markov model (HMM) profiles from 4,934 spider-specific core ortholog groups ([9]). After translating putative open reading frames (ORFs) from each of the six transcriptomes into protein sequences using Trinity’s TRANSDECODER utility, we searched the spider ortholog group set against our translated proteins. We then calculated the proportion of total HMMs that successfully matched at least one sequence in each of the assembled transcriptomes (at a max e-value threshold of 1 x 10^−10^). Finally, we measured the percent of total gene length that was captured by each assembled transcript using the “ortholog hit ratio” (OHR; [53]). This metric compares the length of an assembled transcript to the full length of its top Blast hit. Under this method, an OHR close to zero indicates a poorly assembled transcript, an OHR of one indicates that the full length of the gene was captured, while an OHR greater than one suggests the presence of one or more insertions in the transcript. In order to calculate OHR values, XML output files containing hits to the NCBI’s non-redundant (hereafter nr) protein database (see below) for each species were processed with the ortholog_hit_ratio.py script ([54]), which divides the length of the hit in the query sequence by the total length of the subject sequence.

### Functional annotation

To annotate assembled sequences, standalone Blastx searches of the nr protein database were performed using a maximum e-value threshold of 1 x10^-5^, a high scoring segment-pair (HSP) length cutoff of greater than 33 and 20 maximum hits per sequence. Blastx results were then imported into Blast2GO (B2G; [55]), GO terms were mapped from Accession IDs, and sequences with at least one significant hit were annotated with GO terms using an e-value hit filter of 1x10^-6^, an annotation cut off of 55 and a GO weight of 5. In addition, each transcriptome was annotated with GO terms using B2G’s InterProScan ([56]) option, which assigns functional information to sequence data using protein domain and motif information. Each set of annotations was reduced to include a more manageable subset of higher level GO terms by mapping the annotations to a generic GO Slim ontology using the GO-Slim function.

### Venom sequence analysis

To identify potential venom-associated proteins, we downloaded the Arachnoserver spider toxin database ([57]) and inferred homology between the sequences in this database and ORFs from each transcriptome. Blastx hits with > 50% sequence identity and query coverage as well as e-values less than 1 x10^-15^ were considered for further analysis. To this dataset we added successfully annotated sequences whose top blast hit to the nr protein database consisted of a toxin or venom protein. The translated polypeptide sequences corresponding to this list of transcripts were input into the Arachnoserver’s SpiderP utility to look for evidence of signal peptides and propeptides. If one or both of these were found to be present in a sequence with homology to a known venom protein, this was interpreted as evidence that the transcript is a venom gene. Further functional investigation of these putative venom genes was accomplished using an enrichment analysis in B2G. GO terms present in successfully annotated venom genes were compared against the entire set of functionally annotated transcripts using Fisher’s exact test to look for GO terms that are enriched in transcripts likely to function in venom production. P-values were corrected for multiple testing using Benjamini & Hochberg’s false discovery rate (FDR) method ([58]).

### Identification and alignment of orthologous genes

A set of translated ORFs from each assembly was input into InParanoid ([59,60]), which we used to infer orthologs among each pairwise combination of species. This method identifies orthologous coding sequence pairs based upon similarity scores from reciprocal protein Blast searches. Pairwise orthologs were then grouped into clusters containing one gene from each of the six species using QuickParanoid (available from http://pl.postech.ac.kr/QuickParanoid/). Occasionally, multiple isoforms from the same gene were present in a cluster. In such cases, all but one isoform was removed, leaving only one coding sequence per species in each cluster. Because InParanoid identifies orthologs as well as in-paralogs (i.e., genes that duplicated in one species following a speciation event; [60]), clusters containing more than one non-isoform sequence from any single species were systematically removed from the dataset and were not used in subsequent analyses.

The accuracy of downstream analyses can be negatively impacted by multiple sequence alignment (MSA) errors ([61–64]). Because of this, particular care was taken to minimize such errors. First, we used the phylogeny-aware aligner PRANK v.150803 ([65]) to produce a codon-based MSA for each set of orthologs. Next, each PRANK alignment was processed using GUIDANCE2 v.2.0.1 ([66]) with default settings to detect and remove unreliable alignment positions. Each alignment then received another round of filtering to remove gaps and poorly conserved position using Gblocks v.0.91b ([67]). As a final quality control measure, any MSAs that exhibited 60% pairwise identity, 40% identity across all sequences, or that were 100 bp in length were removed.

### Tests for positive selection

To identify genes under positive selection, we estimated rates of substitution for each ortholog alignment using the codeml program in PAML v4.9a ([68]). Tests for positive selection were implemented using the branch-site model ([69,70]); this method allows *ω*—the ratio of the rate of non-synonymous substitutions per non-synonymous site (Ka) to the rate of synonymous substitutions per synonymous site (Ks)—to vary both among different lineages and at different sites in the sequence. A value of *ω <* 1 is commonly accepted as evidence of purifying selection, while a Ka/Ks value greater than one may indicate positive selection [71]). A phylogeny representing the well-established relationships among the six species (e.g., [9]) was used in all selection analyses. The branch-site analyses required specification of a “foreground” branch in which *ω* is allowed to exceed 1, while *ω* is limited to a value ≤ 1 in the remaining “background” branches (Yang 2007). Nine branches, corresponding to each of the six species and each of the three genera, were chosen as foreground branches in successive analyses.

Each alternative model analysis was compared with a null model in which the foreground branch’s *ω* value could not exceed one using a likelihood-ratio test (i.e., 2*(Log Likelihood_HypA_−Log Likelihood_HypB_)). All analyses were replicated three times with different starting values to ensure that likelihood values reached global optima. Likelihood values were compared across replicate runs, and the run with the highest value was chosen for the LRT. *X*^2^ test statistics resulting from LRTs were used to generate p-values, which were corrected for multiple comparisons using the FDR method ([58]) as implemented in the p.adjust function in R v3.0.2 ([72]).

For genes inferred to be under positive selection by the branch-site analysis, we used the Bayes Empirical Bayes (BEB; [69]) approach in codeml to identify the specific amino acids most likely under selection. This method accounts for uncertainty in the Maximum Likelihood estimates of parameters in order to calculate the posterior probability that each amino acid site in a sequence is included in the class of sites with ω > 1. A particular amino acid was considered to have strong evidence for being under positive selection if the BEB posterior probability value was ≥ 0.95. To investigate possible functions of positively selected genes (PSGs), we retrieved GO terms for each of these genes and performed an enrichment analysis using B2G ([55]).

### Orphan gene search

Orphan genes are putative protein-coding sequences that lack homologs in other taxa and may represent examples of lineage-specific adaptive evolution ([10]). Here, orphan genes were identified and validated by comparing potentially protein-coding transcripts that did not successfully hit to sequences in the nr database from one species with similar transcripts in its closely related congener. First, we used TRANSDECODER to find ORFs within transcripts that lacked a significant Blastx hit to the nr protein database. For genes with multiple isoforms, a single exemplar isoform (the longest) was selected for further analysis. The ORFs from each species were then searched using Tblastx against ORFs in their corresponding congeneric transcriptome that similarly lacked Blastx hits. Two transcripts were designated as putative orphan genes if they were reciprocal best hits of one another and the Tblastx results fulfilled the following criteria: a maximum e-value 1 x 10^−10^, a minimum protein hit length of 67 amino acids (201 nucleotides) and minimum total HSP coverage of 40 percent of the query sequence length. The putatively novel genes discovered through this method were then searched against sequences from the two other genera to identify novel transcripts that are shared among a wider range of Entelegyne taxa. GO terms derived from Interproscan were retrieved for sequences identified as lineage-specific genes.

Because the MSA procedure applied to the orthologs is not applicable for pairwise alignments, we implemented an alternative method to align and edit congeneric orphan sequences. Each pair of orphan genes was translated into amino acids using T-Coffee v.11.00 ([73]), and the resulting translated sequences were aligned with M-Coffee ([74]) using the t_coffe_pair, mafft_pair, muscle_pair, and kalign_pair alignment algorithms. We then used T-Coffee’s TCS procedure ([75]) to output a score ranging from 0 to 9 for each alignment position, depending on how consistently that position was aligned by the four different methods. In order to reduce the incidence of alignment errors, we filtered out alignment positions that received a TCS score lower than 8. Nucleotide MSAs were then generated from each filtered amino acid alignment using T-Coffee’s seq_reformat program. As before, Gblocks v.0.91b ([67]) was used to remove gaps and poorly conserved positions from alignments. Only pairwise sequence alignments (PSA) ≥ 100 bp in length and with ≥ 60% identity were used in subsequent analyses.

If many putative orphan genes are actually examples of lineage specific adaptive evolution, we expect *ω* ratios for these sequences to be elevated compared to the genome-wide set of protein coding sequences for which there is no *a priori* expectation of positive selection. To test this, pairwise maximum likelihood estimates of Ka, Ks and *ω* were calculated for each set of congeneric orphan gene alignments using codeml (Runmode = -2). These evolutionary rate parameters were compared with similar estimates derived from each pairwise comparison of congeneric orthologs used in tests for positive selection. Significant differences in Ka, Ks and *ω* between these two datasets were assessed using nonparametric Mann-Whitney U tests implemented with the wilcox.test function in R [72]. We also clustered each set of *ω* estimates that ranged from 0.0 to 1.0 into three bins of equal size (ω ≤ 0.33, 0.33 < ω ≤ 0.67, and 0.67 < ω ≤ 1.0) and designated these as strong, moderate and weak purifying selection, respectively. Although these designations are arbitrary, the differences in the proportion of sequences that fall into each bin reflect variation in the degree to which orphan and ortholog genes are subject to functional constraint. Prior to analysis, unreliable estimates of *ω* due to extremely low (Ks = 0) or extremely high (Ks > 3) sequence divergence were removed from all datasets.

## Results and discussion

### Transcriptome assemblies

Read data for all six species have been submitted to the NCBI Sequence Read Archive (SRA Accession numbers SRX761208, SRX652499, SRX652504, SRX763246, SRX761332, and SRX652508). Assemblies have been deposited to the Dryad Digital Repository (available at http://dx.doi.org/10.5061/dryad.3pq3q). Assembly size ranged from ~41,000 transcripts in *N*. *cooperi* to more than 165,000 in *C*. *vibora* (Table 1). Isoform content appears to be proportionally much higher in *Cicurina* than in other assemblies. The *Cicurina* transcriptomes had the lowest contig N50 scores, while the highest values were associated with the *Nesticus* transcriptomes. The mean and median transcript lengths followed a similar pattern, with *Nesticus* having the highest, *Cicurina* having the lowest and *Habronattus* exhibiting intermediate results (see Table 1). Most reads were successfully mapped back onto the assembled transcripts, though there was variability in the proportions of reads aligned across species. This proportion is highest in *H*. *ustulatus* (~90%) and lowest for *C*. *vibora* (~80%). GC content was generally similar among the different taxa, ranging from 32.97% in *H*. *signatus* to 37.55% in *N*. *cooperi*. GC values are consistent with those of the velvet spider, *Stegodyphus mimosarum*, but lower than the 39.1% GC content seen in the tarantula *Acanthoscurria* ([23]).

**Table 1:** Transcriptome assembly summary statistics.

	*C*. *travisae*	*C*. *vibora*	*H*. *signatus*	*H*. *ustulatus*	*N*. *bishopi*	*N*. *cooperi*
Transcripts	144,351	165,397	55,081	57,456	52,484	41,169
Components (“genes”)	122,700	146,297	53,337	55,642	51,216	35,817
N50	676	418	814	1002	1266	1161
Mean length	519	407	581	640	747	714
Median length	305	276	345	353	396	392
Maximum length	12,541	11,555	11,379	16,104	14,486	14,677
Nucleotides	74,927,623	67,360,479	31,976,435	36,757,749	39,233,450	29,393,279
% GC content	34.99	36.45	32.97	33.46	36.21	37.55
% of reads aligning to transcripts	83.14	79.96	86.01	90.26	89.4	84.91
Mean Read Coverage	22	18	50	52	41	22

We defined read coverage as the number of reads that successfully map to each transcriptomic base pair position. As seen in Table 1, the mean read coverage was highest in the *Habronattus* transcriptomes (~50x) and lowest in the *Cicurina* and *Nesticus cooperi* transcriptomes (18-22x). It should be noted that these values are all underestimates of the true average read coverage because the maximum coverage at any particular nucleotide position was capped at 200x in the Bedtools analysis. The proportion of each transcriptome with a given level of read coverage (summarized in S1 Fig) was relatively similar across *Habronattus* and *Nesticus* taxa. Approximately 90% of the nucleotides in these four transcriptomes have at least 5x coverage, while there is a broader range—36% to 57%—among these taxa for the proportion of total bases meeting the ≥ 20x coverage cutoff, although only in *N*. *cooperi* is this proportion lower than 50%. Coverage levels were markedly lower in *Cicurina*. Only about 66% of the bases found in the *C*. *travisae* transcriptome have coverage depths of 5x or greater, while the proportion of nucleotides with this level of coverage was slightly less than 55% in *C*. *vibora*. The percentage of nucleotides meeting or exceeding the 20x level of read coverage in *C*. *travisae* and *C*. *vibora* is approximately 26% and 20%, respectively. In the context of previously published spider RNA-Seq studies, the range of read coverage levels reported here is neither remarkably high nor unusually small (e.g., [33,76–78]).

Based on the metrics reported in Table 1, the assembly process resulted in assembled transcriptomes that are broadly similar across the *Habronattus* and *Nesticus* taxa—although there are a few notable exceptions seen in *N*. *cooperi*, such as fewer reads, transcripts and lower coverage. With many more transcripts that are often relatively short, however, the *Cicurina* assemblies are in some ways qualitatively different from the other four. Although the transcript quantity and length found in the *Cicurina* assemblies still fall within the range of values seen in other published RNA-Seq studies (e.g., [79,80]), the fact that these assemblies contain a much larger number of transcripts requires explanation. The *Cicurina* transcriptomes include a proportionally larger number of isoforms when compared to other taxa, which could be an indication of greater transcriptional complexity in these species. Alternatively, the large quantity of somewhat shorter transcripts might be an indication of sequence fragmentation. The *Cicurina* transcriptomes were sequenced using 100 bp reads, while the other transcriptomes were sequenced with 50 bp reads. However, it is unclear why longer reads would lead to a greater frequency of fragmented transcripts. A more important factor may be that the verified concentration of *Cicurina* RNA was much lower than in other taxa. In addition, biased base composition—such as AT-rich DNA sequences—is known to cause problems for NGS methods that rely on PCR amplification during library prep, resulting in low coverage and potential assembly difficulties (e.g., [81,82]). Even though all six transcriptomes are AT-rich, it may be the case that a combination of low RNA concentration and high AT content led to lower overall coverage levels for *Cicurina*, resulting in some low-coverage transcripts being assembled into multiple, shorter fragments.

### Quality and completeness assessment

Results of the CEGMA analyses are summarized in Table 2. More than 90 percent of the CEGs are present at least in partial length for four of the six taxa, while the *C*. *vibora* and *N*. *cooperi* assemblies captured 87% and 88% of the CEGs, respectively. These results are broadly consistent with previous CEGMA analyses of spider transcriptomic data ([32,35]), and confirm that all six transcriptomes contain the majority of conserved eukaryotic protein-coding genes. As summarized in S3 Table, searching the spider-specific OGs against translated protein sequences resulted in patterns very similar to those seen in the CEGMA analysis. Species-specific differences aside, our results indicate that most genes in the spider ortholog group dataset are also present in our assemblies.

**Table 2:** Results of CEGMA analyses.

Species	Full-length mapped CEGs (%)	Full-length + partially mapped CEGs (%)
*C*. *travisae*	217 (87.5)	227 (91.53)
*C*. *vibora*	198 (79.84)	216 (87.10)
*H*. *signatus*	222 (89.5)	233 (94.0)
*H*. *ustulatus*	238 (96.0)	243 (98.0)
*N*. *bishopi*	229 (92.34)	242 (97.58)
*N*. *cooperi*	203 (81.85)	219 (88.31)

**Fig 2:**
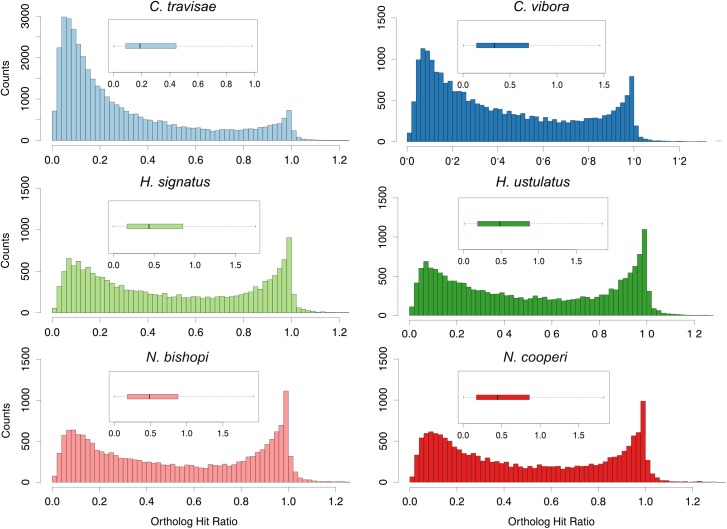
Distribution of OHR values. Histograms (main figure) and boxplots (inset) depict the distribution of OHR values in each species. Outliers have been removed from boxplots to better visualize results. Note the axis scale differences in the *C*. *travisae* plots.

Results of the OHR analyses are shown in Fig 2 and S2 Table. In each species there is a bimodal distribution of OHR values with a peak near 0.0 and an additional peak at 1.0. Despite these similarities, however, the relative sizes of the two peaks and the overall distribution of OHR values differ dramatically in some cases. In particular, the histograms from the two *Cicurina* assemblies show peaks near 0.0 that are larger than the peaks near 1.0. Similarly, the mean and median OHR values are lowest in the *Cicurina* transcriptomes and highest in the *N*. *bishopi* and *H*. *signatus* transcriptomes. These results suggest that a smaller proportion of genes were assembled to full length in the *Cicurina* transcriptomes compared to the other taxa. Despite these larger proportional differences, the number of genes with an OHR greater than 0.5 and an OHR greater than 0.8 was relatively stable across taxa (see S2 Table). It should be noted that the OHR is a conservative approach that may underestimate the percentage of total gene length captured if the phylogenetic distance between the query sequence and its closest hit is great ([54]). Because spider-specific annotated sequences are still sparse in current databases, many transcripts hit to sequences found in distantly related organisms, possibly resulting in OHR values that are lower than they might otherwise be if compared with sequences from closer relatives. Nevertheless, these results suggest that while there are differences in the proportions of fully assembled genes across the six species, each transcriptome successfully captured at least several thousand genes that are approximately full length. Overall, multiple analyses point to the conclusion that the *H*. *ustulatus* and *N*. *bishopi* transcriptomes are the highest quality and most complete assemblies. Although the *Cicurina* assemblies show evidence of fragmentation, the quality and completeness assessment confirms the presence of many full-length protein-coding genes and supports the conclusion that the assembly process was generally successful.

### Functional annotation

Putative homology between transcripts and sequences from the nr protein database was assigned using Blastx. The number of successful hits resulting from the standalone Blastx searches ranged from approximately 39,000 in *C*. *travisae* to about 16,600 in *H*. *signatus*, although these are likely overestimates of the number of genes discovered, since they include, in some cases, isoforms derived from the same coding sequence. The percentage of all transcripts with hits varied from only 14% for *C*. *vibora* to just over 40% for *N*. *cooperi* (S4 Table). There were no fungal taxa among the 100 most frequently hit species, suggesting that fungal contamination was likely not a significant problem in these assemblies.

Despite large variability in transcript quantity and number of Blastx hits, the number of transcripts that were successfully annotated with GO terms was consistent across the six transcriptomes. The highest number was seen in *C*. *travisae* (15,866 transcripts) and the lowest was in *H*. *signatus* (13,477 transcripts; S4 Table). Protein domain information provided by InterProscan resulted in the addition of 377–802 GO terms to sequences that failed to hit any proteins in the nr database. In the *Habronattus* and *Nesticus* transcriptomes, roughly 80% of sequences with Blastx hits were also successfully annotated with GO terms, while this proportion was substantially lower in the *Cicurina* transcriptomes. In particular, only about 40% of the *C*. *travisae* Blastx hits resulted in successful annotations. This suggests that many of these hits were not associated with GO terms and/or that many of the GO terms mapped to the sequences did not pass the annotation filter.

For all species, a majority of assembled transcripts could not be annotated with GO terms, suggesting that many of these transcripts may be sequence fragments, derived from non-coding RNAs, or are spider-specific genes that do not yet have homologs in the nr protein database. A relatively large proportion of sequences without homology to known proteins have been seen in other spider transcriptome studies (e.g., [32,76,78]). As well-characterized spider genomic data continue to be added to existing databases, there will presumably be a concomitant increase in the proportion of newly published spider sequences with homologous matches.

**Fig 3:**
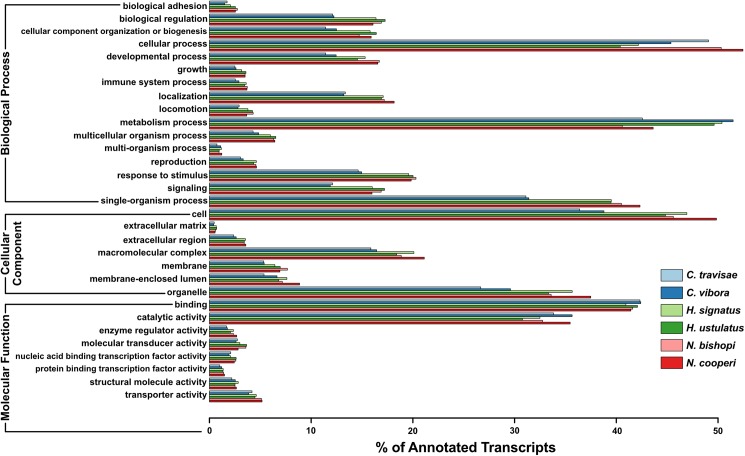
Results of GO analysis. Bar chart showing the proportion of annotated transcripts containing various GO terms (Level 2) across the 3 major categories.

The total number of GO terms assigned to each transcriptome varied from about 64,000 in *C*. *travisae* to nearly 80,000 for *H*. *ustulatus*, although the number of distinct GO terms found in each transcriptome was in the low hundreds (see S4 Table). The fraction of successfully annotated transcripts with GO terms (Level 2) found in the three major categories—biological process, molecular function and cellular component—is depicted in Fig 3. Overall, the relative frequency of different GO terms is consistent across taxa, and with some exceptions, the relative abundance of many of the most common GO terms found (e.g., “cellular” and “metabolic” processes, “binding”, “catalytic activity”, etc.) is similar to findings from previous transcriptomic analyses of a variety of different taxa. These include studies of other spiders (e.g., [31,33,76]), other arachnids (e.g., [8,83,84]), other metazoans (e.g.,[53,85,86]), and even non-metazoans (e.g., [87,88]). One previously suggested explanation for these similarities (e.g., [7]) is that transcriptome assemblies from short-read RNA-Seq analyses are biased toward the capture of highly expressed, conserved housekeeping genes. Alternatively, homology-based methods may be biased toward annotating conserved housekeeping genes, particularly in species for which closely related sequences are largely unavailable in databases.

### Venom genes

Blasting all six transcriptomes against both the nr protein and Arachnoserver spider toxins databases revealed evidence of homology to venom proteins in 146 transcripts. Of these, 101 transcripts were inferred to contain a signal peptide and/or propeptide sequence region. These 101 transcripts were considered to be putative venom protein-coding genes (S6 Table). Eighty-three of these were successfully functionally annotated, and an enrichment analysis identified five GO terms that are overrepresented (S5 Table). Based on the analysis, we found that venom transcripts are approximately 16 times more likely to be localized in the “extracellular region” (i.e., outside of the plasma membrane) than are non-venom transcripts. In fact, “extracellular region” was the only cellular component GO term found in any of the venom transcripts. Other studies that specifically targeted spider venom gland transcripts have also reported significant enrichment of the “extracellular region” term in venom genes (e.g., [28,77]). These venom-related transcripts are also ~ 4-8x more likely to be assigned functions related to peptidase activity—which involves hydrolyzing the peptide bonds connecting amino acids in a polypeptide chain ([89])—cell adhesion, and enzyme regulation.

Some of the toxins most frequently inferred to be homologous to sequences in our transcriptomes include agatoxins, pisautoxins, and aranetoxins. Agatoxins were first isolated from the funnel weaver *Agelenopsis aperta* and are known to induce paralysis in prey items by blocking or otherwise interfering with the normal behavior of ion channels ([90]). First identified in the fishing spider *Dolomedes mizhoanus*, pisautoxins are proteins containing cystine knot motifs. It has been speculated that pisautoxins play a role in blocking calcium ion channels and/or inhibiting P2X3 receptors, but these functions have not been experimentally verified ([27]). Aranetoxins are a group of venoms that were first isolated from the Chinese orbweaver, *Araneus ventricosus*, whose specific molecular functions have yet to be elucidated ([91]). A fourth common venom protein is Techylectin-1-Phoneutria, which is a unique Ctenitoxin known only from the Brazilian armed spider *Phoneutria nigriventer* ([92]). This protein exhibits high sequence identity (> 50%) to the techylectin-5A protein found in the Japanese horseshoe crab, although the latter protein is not known to play a toxin-related role ([93]). Little is known about the biological activity of Techylectin-1-Phoneutria other than that it appears to be a carbohydrate-binding lectin with a fibrinogen C-terminal domain ([92]).

Based upon available data, it is not clear whether the frequencies of these particular venom families are primarily a reflection of the actual diversity of the toxins produced by *Cicurina*, *Habronattus* and *Nesticus* spiders or simply a product of biases in the taxonomic composition of the relatively small Arachnoserver database. While the homology-based inference of venom proteins presented here is suggestive, it would be unwise to draw definitive conclusions regarding these results until the functions of these putative venom genes can be validated by more targeted and perhaps experimental approaches.

### Ortholog identification & tests for positive selection

The Transdecoder open reading frame prediction identified between 13,000 and 17,000 unique ORFs in each species. Using these coding sequences as input, the ortholog inference and clustering procedures implemented in InParanoid and QuickParanoid resulted in the discovery of 3,421 different clusters containing one gene from each of the six transcriptomes. For further analyses, each cluster was treated as a group of orthologous protein-coding genes. After implementing MSA and quality filtering procedures, 3,345 alignments remained, with a median gap-free length of ~851 bp (range of 105–7038 bp).

**Fig 4:**
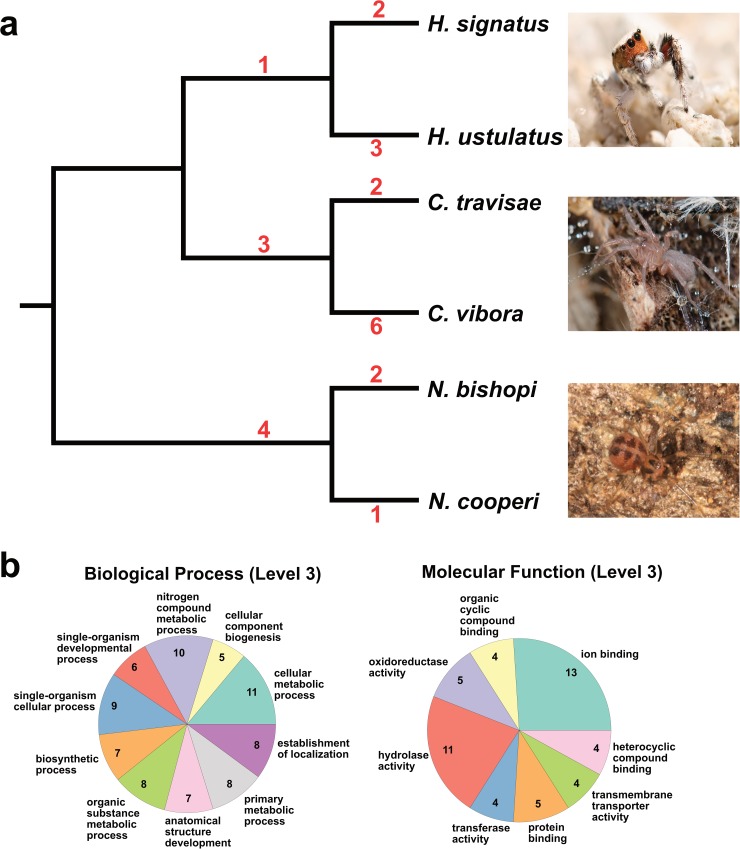
Tests for positive selection. (a) Phylogeny indicating the number of instances in which each branch was found to be under positive selection. *Nesticus* image used under a CC BY license with permission from Alan Cressler. (b) Most frequent GO terms found in PSGs.

Using these 3,345 alignments, we tested for positive selection along each genus- and species-level branch of the canonical six species phylogeny using codeml’s branch-site model. Replicate runs with different starting values for *ω* and *κ* (the transition-transversion ratio) generally resulted in very similar likelihood scores, but when the scores differed across replicates, the run with the higher score was used. After the FDR correction for multiple testing, LRTs between null and alternative models returned statistically significant (at a FDR of 0.1) signatures of positive selection along 24 branches from 22 ortholog alignments (Table 3). The BEB analysis found that 20 of these putative PSGs contained at least one codon belonging to the class of codons under positive selection at a posterior probability ≥ 0.95. On average, each PSG contained ~ four amino acids under positive selection. When these positively selected regions were present, they made up, on average, only 4.9% of the entire aligned sequence length. As expected, these results suggest that even in genes undergoing adaptive evolution, most amino acids are likely to be under strong purifying selection. The frequency of positive selection on each branch of the phylogeny is depicted in Fig 4A. Overall, instances of positive selection were relatively rare. Although PSGs are not heavily concentrated in any specific lineage, a slightly higher number are found on *Cicurina* branches. The *Cicurina* species, which are restricted to deep cave environments with high humidity and total darkness, also exhibit a higher degree of ecological specialization than the other taxa studied.

**Table 3:** Genes under positive selection as identified by codeml branch-site analysis.

Branch	Putative Protein ID	ω	FDR	Proportion under selection
*Cicurina*	Myosin heavy chain, muscle-like	17.8	4.48E-05	0.015
*Nesticus*		87.9	6.58E-02	0.009
*Habronattus*	Calcium-transporting ATPase sarcoplasmic/endoplasmic reticulum type	107.9	4.61E-03	0.014
*Nesticus*		314.8	4.36E-02	0.018
*H*. *ustulatus*	Alpha-actinin, sarcomeric	999	1.59E-06	0.008
*C*. *travisae*	Calcium homeostasis endoplasmic reticulum protein	114.5	1.03E-04	0.024
*C*. *travisae*	Peroxidasin	74.3	4.99E-03	0.036
*N*. *bishopi*	Hemocyanin G	999	4.53E-10	0.048
*C*. *vibora*	Homothorax 1	999	1.59E-06	0.018
*Cicurina*	Clotting factor B	267.6	4.52E-02	0.059
*N*. *cooperi*	S-adenosylmethionine synthase	999	1.68E-03	0.027
*C*. *vibora*	mRNA cap guanine-N7 methyltransferase	999	9.31E-02	0.012
*H*. *signatus*	Zinc finger protein 226	44.4	1.55E-02	0.028
*C*. *vibora*	tRNA (adenine(58)-N(1))-methyltransferase non-catalytic subunit TRM6-like	999	4.48E-05	0.017
*Nesticus*	Tropomyosin	152.9	4.36E-02	0.051
*H*. *signatus*	Ubiquitin-conjugating enzyme E2 R2	999	1.63E-02	0.033
*H*. *ustulatus*	Ras-related protein Rab-5C	696.5	4.48E-05	0.126
*H*. *ustulatus*	Lysozyme 1	999	7.80E-02	0.08
*N*. *bishopi*	L-azetidine-2-carboxylic acid acetyltransferase	999	4.52E-02	0.081
*Cicurina*	U24-ctenitoxin-Pn1a	26.7	8.75E-02	0.1
*C*. *vibora*	Mitochondrial fission factor	999	1.98E-02	0.011
*C*. *vibora*	Protein boule-like	999	4.52E-02	0.035
*Nesticus*	WD repeat-containing protein mio-B	227.5	4.52E-02	0.315
*C*. *vibora*	Eukaryotic translation initiation factor 4E-binding protein 1	999	4.93E-02	0.021

Of the 22 ortholog alignments with evidence for positive selection, all but one were successfully annotated with GO terms. Many of the most frequent terms under the biological process category relate to cellular, metabolic, single-organism cellular, developmental processes, and localization (Fig 4B); likewise, the common terms under the molecular function category include various types of binding, as well as “hydrolase”, “oxidoreductase” and “transferase” activity. These latter three terms refer to the catalysis of various biochemical reactions, including hydrolysis, oxidation-reduction reactions, and the transfer of different chemical groups (e.g., methyl) from one compound to another ([89]). Owing perhaps to the small size of the selection dataset, Fisher’s exact test did not find statistically significant evidence that GO terms were overrepresented in PSGs.

Although the homology-based gene identifications should be treated cautiously until more data are available, some tentative inferences can be made regarding the functions of genes under positive selection. One of the most striking patterns seen among the PSGs (listed in Table 3) is the presence of several proteins involved in muscle contraction, including myosin, actin and calcium ATPase. The codeml analysis detected selection on the myosin heavy chain on both the *Cicurina* and *Nesticus* branches. This protein is assembled into the backbone of muscle thick filaments and is responsible for controlling muscle contraction ([94]). In addition, we found evidence for selection on alpha-actin on the *H*. *ustulatus* branch. This protein is the major constituent of muscle thin filaments, which, combined with the myosin-dominated thick filaments, form sarcomeres ([95]). Another apparent muscle-related protein under selection in multiple lineages—in this case, *Habronattus* and *Nesticus*—is the sarco/endoplasimic form of calcium-transporting ATPase, which is responsible for removing calcium ions from the cytosol after muscle contraction ([96]). Tropomyosin, another protein involved in muscle contraction (although there are non-muscle forms as well; [97]), was found to be under selection in *N*. *bishopi*.

Other noteworthy PSGs include the appendage development gene *homothorax*-1 ([98]), the gene encoding respiratory protein hemocyanin G ([99]), and *Boule*, a gene known to be involved in metazoan gametogenesis ([100,101]). *Boule*, unlike many other reproductive proteins has previously been found to evolve primarily under purifying selection in other taxa ([102]), thus is it somewhat surprising to find it under positive selection in our data. We also found evidence in the *Cicurina* lineage of positive selection acting on a likely venom protein with high sequence similarity to the U24-ctenitoxin-Pn1a protein first sequenced from *Phoneutria nigriventer*. Although information is limited, this toxin is thought to be an inhibitor of cysteine proteinase activity ([92]).

In total this analysis identified evidence of adaptive molecular evolution in only 0.08% of the 29,835 (3,315 total ortholog alignments x nine lineages) branches examined. In comparison with several recent genome-wide scans for positive selection (e.g., [103–105]), our results suggest that a much smaller proportion of entelegyne spider genes have evolved under positive selection. The only other comparable study of spider genomics for a large number of loci found evidence of positive selection in a similarly low proportion of sequences ([34], but also see [35,36]). Although it is possible that our results may reflect the actual prevalence of positive selection in some entelegyne spider species, there are likely additional factors that contributed to the small number of inferred PSGs.

Our MSA strategy utilizing PRANK and GUIDANCE is a conservative approach that has been shown to reduce false negatives, albeit with a concomitant decrease in power to detect true positives ([106]). Preliminary analyses using different MSA procedures resulted in substantially larger numbers of inferred PSGs (data not shown), likely false positives resulting from alignment problems. Another factor that may have reduced the incidence of detected PSGs in our analysis is the sparse sampling of relatively divergent taxa. Previous work ([107]) has shown that the branch-site test has difficulty detecting positive selection along branches with saturated synonymous substitutions. Although observed divergence levels among congeneric species pairs was low (median Ks = 0.0105), the three genera are distant relatives ([9]), resulting in much higher divergences levels in interior branches (median Ks = 1.4036). Thus, it may be the case that our analysis missed some instances of adaptive protein evolution—especially those that occurred along longer interior branches—due to saturation. More broadly, other researchers (e.g. [108–110]) have found that power to detect positive selection on a particular phylogenetic branch increases with the addition of taxa. Given our limited sample size, it is also possible that the analysis lacked statistical power to detect positive selection events occurring on the phylogeny. Finally, another important factor likely driving down the incidence of observed adaptive evolution may have been our choice to examine only single-copy ortholog groups. Although other similar studies have found higher rates of positive selection among single-copy orthologs ([103–105]), it may be the case that these gene families are subject to lower rates of positive selection than multi-copy gene families. As more large-scale scans for positive selection are performed on spider genomic data, it will be important to see whether or not the pattern of low rates of positive selection in spider lineages seen here and in previous work is maintained.

### Orphan genes

**Fig 5:**
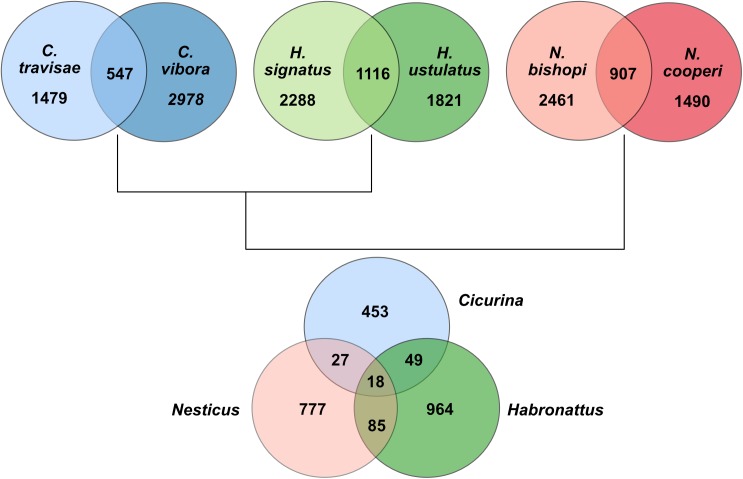
Venn diagrams showing results of the orphan gene search. Numbers within circle intersections represent genes shared among taxa. Numbers outside of intersections are genes exclusive to that taxon.

After removing isoforms and filtering out sequences without an ORF at least 200 bp in length, each transcriptome still included more than 2,000 potentially coding sequences without hits to the nr database. Using a paired congeneric sampling strategy, we verified the presence of lineage specific orphan genes under the assumption that a previously unknown sequence with coding potential is less likely to be the result of a sequencing error or assembly artifact if also present in a close congener. The results of this search are summarized with Venn diagrams in Fig 5. The *Cicurina* transcriptomes shared 547 putatively novel genes; the *Habronattus* transcriptomes shared 1,116 novel genes, and the *Nesticus* taxa shared 907 putative orphan genes. When comparing shared genes across different genera there was a steep drop-off in the number of undescribed genes found in common (Fig 5); in total, we found evidence for 18 novel genes shared among all taxa.

The fact that *Cicurina* and *Habronattus*, which are sister taxa in this analysis, share fewer taxonomically-restricted genes than do *Habronattus* and *Nesticus* suggests that variation in transcriptome completeness (e.g., due to variability in the number of samples, sex and tissue types used) likely influenced the number of shared genes discovered. However, the lack of orphan genes in one transcriptome that are present in another is, in some cases, likely a reflection of true absence. Given the finding that orphan genes comprise ~10–20% of each newly sequenced genome (e.g., [111]), it is not unreasonable to conclude that many of the orphans found only in congeners are unique to those individual lineages. The taxa used in this analysis, while all entelegyne spiders, are nonetheless only distantly related. In fact, the divergence time between *Nesticus* and *Cicurina+Habronattus* (~154–290 MA; [9]) is roughly akin to the split between humans and monotremes. As such, it is likely that many of the genus-specific orphan genes detected in this analysis evolved *de novo* after the different lineages diverged from their respective common ancestors. Also, differences in the quantity of orphan genes seen among lineages might be influenced by lineage-specific losses of orphan genes (e.g., [112]).

Finally, another factor that undoubtedly contributed to the relatively small number of orphan genes shared among all transcriptomes were the specific quality control filters we chose. In preliminary analyses, using fewer and less stringent filters resulted in approximately 4-6x more taxonomically-restricted genes shared among genera as well as among all species. After exploring a number of different possible filtering parameters, the results reported here represent our best compromise between sensitivity and reliability. However, it is reasonable to presume that many true orphan genes were missed simply due to our choice of filters.

Because the novel genes discovered by this analysis lacked—by definition—hits to the nr database, the vast majority of them could not be functionally annotated. Nevertheless, each comparison between congeneric pairs revealed sequences to which GO terms could be applied using protein domain and motif information from InterProScan. Functional annotations were assigned to 56 *Cicurina*, 34 *Habronattus*, and 76 *Nesticus* orphans, respectively. Under the biological process category, the most frequent Level 2 terms were “cellular process”, “single-organism process” and “metabolic process”. An examination of more specific functions (GO Level 3 and above), suggests that many of these transcripts encode gene products with metabolic roles; 13 of the 19 most frequent terms involve metabolic processes (S2A Fig). Under the molecular function category, the two most common Level 2 terms are “binding” (85 transcripts) and “catalytic activity” (19 transcripts). At more specific GO levels, the vast majority of the most common terms relate to various forms of binding, including ion binding, protein and DNA binding (S2B Fig), suggesting that these are functions shared in common by many of the orphan genes. Overall, the GO terms assigned to orphan genes are qualitatively very similar to the most frequent GO terms found in all the annotated transcripts. Based on this, it seems likely that the functional annotations assigned by InterProScan were heavily biased toward highly conserved protein domains, making it difficult to infer what, if any, unique spider-specific roles these orphan genes perform.

**Fig 6:**
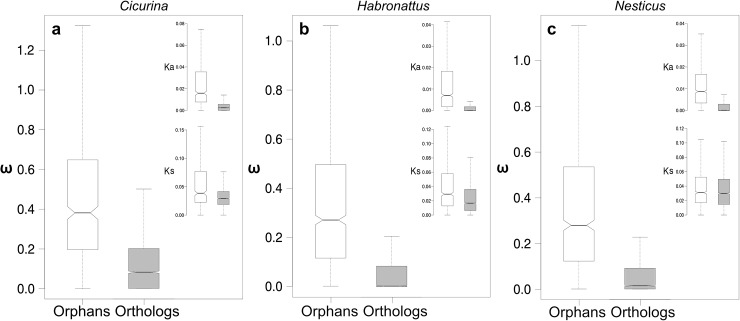
Boxplots of evolutionary rate comparisons between orthologs (grey) and orphan genes (white). (a) *Cicurina*, (b) *Habronattus* and (c) *Nesticus*. The main plot shows estimates of *ω*, while the inset shows Ka (top) and Ks (bottom). Outliers have been removed to better visualize the inter-quartile ranges. Mann-Whitney U tests indicate that all parameter estimates are significantly greater in orphan genes, with the exception of the *Nesticus* Ks comparison which shows no significant difference.

After aligning and quality control filtering, we estimated substitution rates for 499 *Cicurina* orphan genes, 1104 *Habronattus* orphans, and 893 *Nesticus* orphans. Median aligned orphan lengths ranged from 408 bp for *Cicurina* to 510 bp for *Habronattus*, consistent with previous results showing that taxonomically restricted genes tend to be shorter than phylogenetically older sequences (e.g., [112,113]). The results of comparing Ka, Ks and *ω* between congeneric orphan genes and the congeneric orthologs are summarized in Fig 6 and Table 4. In nearly every case, estimates of all three evolutionary rate parameters showed a consistent and statistically significant trend toward larger values in the orphans than in the orthologs (Mann-Whitney U tests, P < 2.2 × 10^−16^ suggesting an increase in the overall rate of evolution in the orphan genes. The only exception is the comparison of Ks values in the *Nesticus* sequences, where orthologs have a slight and non-significantly higher rate of synonymous substitutions (Mann-Whitney U test, P = 0.1389). Of particular importance, median estimates of *ω* ranged from ~ 4.7x to more than 200x larger in the orphan genes than in the orthologs. This high latter value is primarily a reflection of the especially small number of amino acid replacements in the *Habronattus* orthologs. In principle, this result could be explained by reduced synonymous substitution rates in the orphans. However, because Ks values were actually higher in most of the orphan genes, the concomitant increase seen in *ω* ratios is necessarily a result of excess nonsynonymous substitutions. It should be noted that we repeated this statistical analysis without first removing the most divergent sequences (Ks > 3), which did not change the overall outcomes reported here.

**Table 4:** Comparison of substitution rates between congeneric orphan genes and orthologs.

	*Cicurina*		*Habronattus*		*Nesticus*	
	Orphans	Orthologs	Orphans	Orthologs	Orphans	Orthologs
Median ω	0.380	0.081	0.257	0.001	0.276	0.017
Median Ka	0.016	0.002	0.008	0	0.009	8E-04
Median Ks	0.039	0.029	0.033	0.021	0.032	0.032
ω ≤ 0.33 (%)	42.20	88.39	59.42	95.68	55.43	95.22
0.33 < ω ≤ 0.67 (%)	32.97	8.41	23.70	3.61	25.89	3.80
0.67 < ω ≤ 1.0 (%)	10.77	1.91	9.81	0.56	8.91	0.68
ω > 1.0 (%)	14.1	1.29	7.1	0.15	9.77	0.29

This discovery—elevated *ω* ratios driven by increased rates of nonsynonymous substitutions—is consistent with the hypothesis that orphan genes are involved in lineage-specific adaptive processes (e.g., [10,114,115]). However, positive selection is not the only potential explanation for this pattern. To some extent, our results might also be explained by a relaxation in purifying selection against the fixation of deleterious amino acids in orphan genes. For example, fewer orphans appear to be evolving under strong purifying selection as compared to the orthologs, and a larger fraction of the orphans show rates of substitution more consistent with weaker purifying selection or even neutral evolution (Table 4**).** Given this, the elevated rates of nonsynonymous substitutions could suggest relaxed functional constraints or even incipient pseudogenization in some of the orphans. In particular, if single-copy orthologs tend to experience stronger than average purifying selection (e.g., [116]), this may have the effect of inflating the disparity in ω ratios seen between our ortholog and orphan gene datasets. However, most of the orphans appear to be evolving at rates primarily indicative of moderate-to-strong purifying selection (Table 4**)**, consistent with previous studies of orphan genes (e.g., [112]). This suggests that a decrease in the extent of purifying selection is unlikely to be the only factor driving the higher frequency of amino acid substitutions.

Importantly, the total proportion of comparisons in which we found pairwise congeneric estimates of *ω* > 1 is more than an order of magnitude greater in the orphans (9.54%) than in the orthologs (0.60%). This result strongly suggests an increased role for positive selection in orphan sequence evolution. Indeed, visual inspection of orphan alignments reveals many striking cases in which the number of amino acid substitutions greatly outnumber silent site substitutions in otherwise relatively conserved, high quality alignments. If relaxed selection alone were driving the observed increase in the rate of nonsynonymous substitutions, we would not expect Ka to exceed Ks in so many instances. Although these results are suggestive and in line with previous work finding evidence of adaptive evolution in orphans (e.g., [113,115,117]), we cannot definitively conclude that all orphan genes with *ω* > 1 have indeed undergone positive selection. This is because the tests for positive selection that we performed on the ortholog dataset are not applicable for pairwise sequence comparisons. This important caveat aside, our results point to a plausible conclusion that a substantial proportion of these lineage-specific spider genes have likely experienced bouts of adaptive protein evolution.

As genomic resources for non-model organisms have become more readily available, interest in the function, origin, and evolutionary dynamics of taxonomically restricted genes has increased (e.g., [11,111]). We believe that the orphan genes identified in this analysis represent fertile ground for future explorations aimed at understanding molecular evolution in entelegyne spiders. In particular, analysis of expression patterns in orphans should help substantiate the biochemical validity of these sequences and provide much needed information regarding their functional roles. In addition, we assert that the congeneric pair strategy implemented here is an effective means of identifying and validating new lineage-specific genes.

## Supporting information

S1 Table RNA extraction and sequencing information. (XLSX)

S2 Table Results of Ortholog Hit Ratio analyses. (XLSX)

S3 Table Matches to spider core ortholog dataset. (XLSX)

S4 Table Blastx and B2G functional annotation results. (XLSX)

S5 Table Results of enrichment analysis, showing GO terms overrepresented in venom-related transcripts. (XLSX)

S6 Table List of putative venom proteins found in the six transcriptomes. (XLSX)

S1 Fig Proportion of each transcriptome with ≥ 5x, ≥ 10x and ≥ 20x read coverage. (TIFF)

S2 Fig **Distribution of the most common GO terms assigned to orphan genes in the (a) biological process and (b) molecular function categories.** Only GO terms at levels 3 and above present in at least ten transcripts are shown.
(TIFF)
