# Supplementary figures and images for: Comparative transcriptomics of Entelegyne spiders (Araneae, Entelegynae), with emphasis on molecular evolution of orphan genes
Source: PLoS One. 2017 Apr 5;12(4):e0174102. doi: 10.1371/journal.pone.0174102 (PMC5381867; doi:10.1371/journal.pone.0174102)

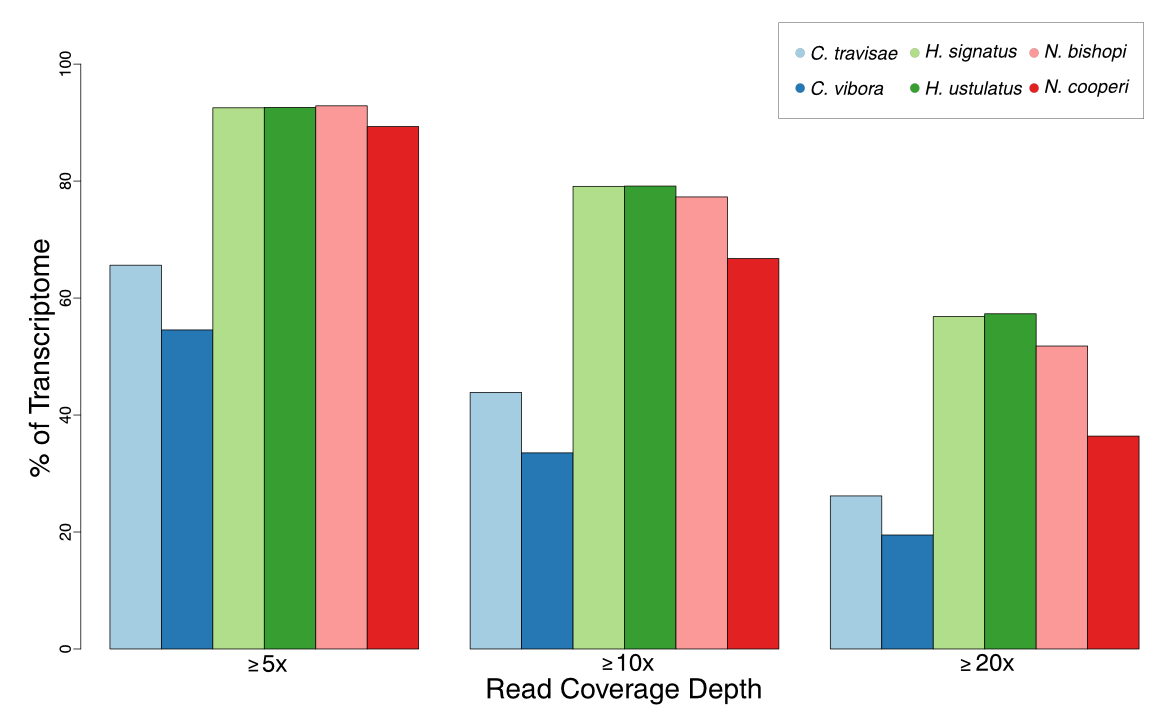

Supplement: S1 Fig — (TIFF) [file pone.0174102.s007.tiff]

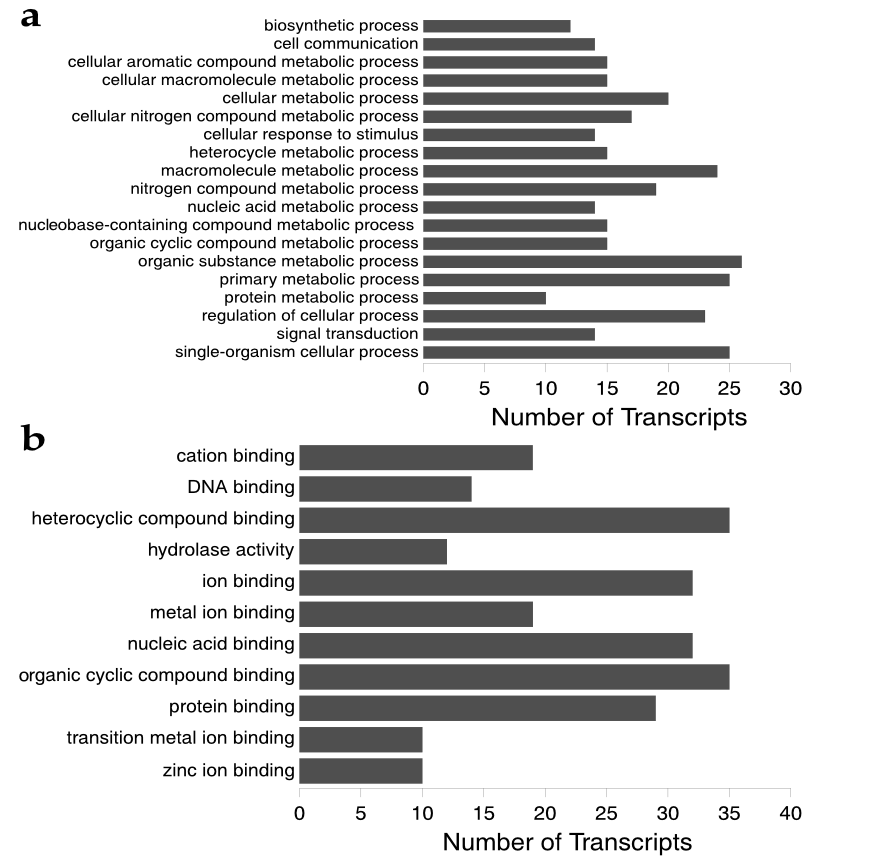

Supplement: S2 Fig — Distribution of the most common GO terms assigned to orphan genes in the (a) biological process and (b) molecular function categories. Only GO terms at levels 3 and above present in at least ten transcripts are shown. (TIFF) [file pone.0174102.s008.tiff]
